# HEALTH CARE PRIORITIES OF OLDER KOREAN IMMIGRANTS: THROUGH THE LENS OF LIFE COURSE THEORY

**DOI:** 10.1093/geroni/igz038.3269

**Published:** 2019-11-08

**Authors:** Hyeyoung K Park, Sharron L Docherty, Cristina C Hendrix, Ruth A Anderson, Kimberly S Johnson

**Affiliations:** 1 University of Massachusetts Amherst College of Nursing, Amherst, Massachusetts, United States; 2 Duke University School of Nursing, Durham, North Carolina, United States; 3 University of North Carolina at Chapel Hill School of Nursing, Chapel Hill, North Carolina, United States

## Abstract

More than half of Korean Americans living in the US are immigrants, these immigrants hold unique cultural perspectives, including collectivism and filial piety that originates from Korean culture. Every older adult has life experiences and background that build and shape their own wishes and values for their health care goals. Thus, a qualitative descriptive study was conducted using the Life Course Theory as a guiding framework to examine older Korean immigrants’ health care goals and the influence of their life courses. Twenty six interviews from 13 participants were analyzed using content thematic analysis. Study rigor was ensured by audit trail, peer debriefing, and prolonged engagement. Data were organized under five overarching themes: health care priorities, time, location, linked lives, and turning point. Older Korean immigrants valued painlessness and being independent as health care goals (Health care priorities). They experienced a dynamic historical period in Korea before immigrating to the US (Time). Once they reached the US, they were disconnected from their social support and traditional values (Location). Children and Korean churches constitute older Korean immigrants’ primary support system once in the US (Linked lives). Their tumultuous life experiences contributed to their current perspectives on health care goals and priorities (Turning point). In studies of older immigrant populations, it is important to acknowledge individual differences while simultaneously understanding the general life history and cultural background behind individuals’ values and perspectives. Life course approach provides both a contextual understanding of older adults’ backgrounds and the trajectories of their individual life courses.

